# CD59 mediates cartilage patterning during spontaneous tail regeneration

**DOI:** 10.1038/srep12798

**Published:** 2015-08-04

**Authors:** Xue Bai, Yingjie Wang, Lili Man, Qing Zhang, Cheng Sun, Wen Hu, Yan Liu, Mei Liu, Xiaosong Gu, Yongjun Wang

**Affiliations:** 1 Key Laboratory of Neuroregeneration, Co-innovation Center of Neuroregeneration, Nantong University, Nantong 226001, PR China

## Abstract

The regeneration-competent adult animals have ability to regenerate their lost complex appendages with a near-perfect replica, owing to the positional identity acquired by the progenitor cells in the blastema, i.e. the blastemal cells. CD59, a CD59/Ly6 family member, has been identified as a regulator of positional identity in the tail blastemal cells of *Gekko japonicus*. To determine whether this function of CD59 is unique to the regenerative amniote(s) and how CD59 mediates PD axis patterning during tail regeneration, we examined its protective role on the complement-mediated cell lysis and intervened CD59 expression in the tail blastemal cells using an *in vivo* model of adenovirus transfection. Our data revealed that gecko CD59 was able to inhibit complement-mediated cell lysis. Meanwhile, CD59 functioned on positional identity through expression in cartilage precursor cells. Intervening positional identity by overexpression or siRNA knockdown of CD59 resulted in abnormal cartilaginous cone patterning due to the decreased differentiation of blastemal cells to cartilage precursor cells. The cartilage formation-related genes were found to be under the regulation of CD59. These results indicate that CD59, an evolutionarily transitional molecule linking immune and regenerative regulation, affects tail regeneration by mediating cartilage patterning.

The ability to regenerate appendages or organs is almost entirely lost in adult mammals during evolution. Several classes of vertebrates, including fish, amphibians and reptiles, still retain the robust ability to regenerate limbs, tail, jaws, lens, or (and) small sections of the heart^1^,^2^,^3^,^4^,^5^. These extant regenerative models, with undefined cellular and molecular mechanisms to control regenerative ability or rate of regeneration, have provided potential possibility for regenerating the appendages or organs of adult mammals in the future. A successful regeneration needs to be of high fidelity such that urodele limb amputated from wrist regenerates a hand, whereas from shoulder regenerates an entire arm^6^. It is well known that the reconstruction of limb and tail occurs from a proliferative zone, the blastema, in which progenitor cells are dedifferentiated from internal tissues or migrated from satellite cells^7^. The blastema keeps positional identity, which is used to regenerate only correct elements along proximo-distal (PD) axis^8^. Previous experiments have shown that altering the positional information of limb blastemal cells by retinoic acid (RA) resulted in abnormal limb regeneration^9^.

The heterogeneous blastemal cells have shown a lineage-restricted differentiation during the limb or tail regeneration, exhibiting strong memories of their tissue origins^8^,^10^. This observation leads to the assumption that blastemal cells deriving from different tissues all harbour memory features. However, evidences from cartilage and Schwann-derived blastemal cells indicate that precursor cells of cartilage have PD positional identity while the latter do not, suggesting that positional identity is tissue specific^8^. Pioneering work from the Brockes’ group discovered that Prod1, a protein from the CD59/Ly6 family, regulated PD cell identity in amphibian limb regeneration^11^,^12^,^13^. Certain vertebrate lineages lacks regenerative ability partly because the absence of this gene^14^.

Several species from non-mammalian amniotes, such as gecko and lizard, are able to undergo scar-free wound healing and regeneration following tail amputation^15^,^16^. A conserved morphological and histological program, including wound healing, blastema formation and differentiation, and the regeneration of major axial structures, occurs in these amniotes^3^,^4^. Most strikingly, the regenerated tail reconstructs an unsegmented cartilage tube, instead of the vertebrae of the original tail. At the early stage of the tail regeneration, the extreme proximal cartilage tube in direct contact with the most terminal vertebra of the original tail undergoes endochondral ossification. While at the later stages, the distal cartilage tube mineralizes without endochondral ossification^17^. Similar to those of amphibian limb, the blastemal cells of gecko tail have established positional information to re-specify the PD axis before differentiating to various tissues^18^. CD59, another member from the CD59/Ly6 family, was found to regulate PD cell identity during the gecko tail regeneration^18^. CD59 protein is better known as a major inhibitor of complementary activation by virtue of its ability to bind to C5b-8 and C5b-9 complexes, thus preventing the formation of polymeric C9^19^. While CD59 is normally attached to the cell surface via a glycosylphosphatidylinositol (GPI) anchor, it also exists in a number of soluble forms in saliva, amniotic fluid and urine, etc^20^. It is unclear whether the function of CD59 to mediate positional identity is unique to the regenerative species. It also remains to be determined how CD59 regulates the PD axis patterning. Here, we established an *in vivo* model of adenovirus vectors intervention, which enabled us to examine CD59-mediated blastemal cells and the patterning of gecko tail. Our results demonstrate that in the regenerative gecko, CD59 plays important roles for both positional identity and immune regulation. We showed that CD59 was involved in cartilage patterning by affecting the differentiation of blastemal cells during tail regeneration.

## Results

### Expression of CD59 in the early regenerating tail

**Figure 1:**
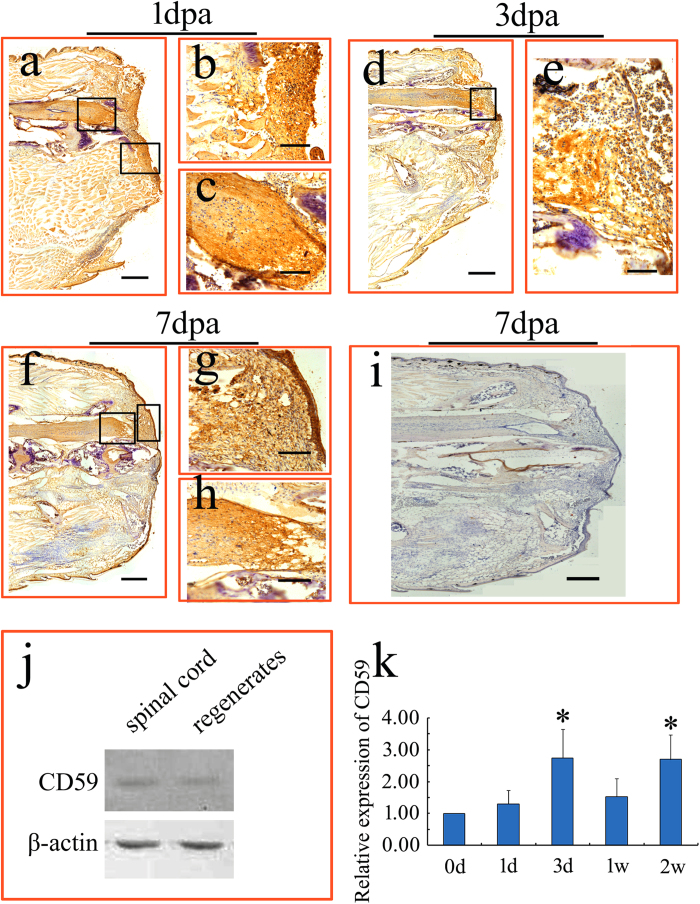
Tissue expression of CD59 in the regenerates following gecko tail amputation. (**a**–**c**) Expression of CD59 in the tail stump at 1 dpa. (**b**) and (**c**) indicate higher magnification of the boxed regions in (**a**); (**d**) Expression of CD59 in the tail stump at 3 dpa; (**e**) indicates higher magnification of the boxed region in (**d**); (**f**–**h**) Expression of CD59 in the tail stump at 1 wpa. (**g**) and (**h**) indicate higher magnification of the boxed regions in (**f**); (**i**) indicates negative control without primary antibody; (**j**) Western blot of tissues from spinal cord and regenerates at 1 dpa; (**k**) Real-time PCR analysis of *CD59* expression in the tail stumps at 0 dpa, 1 dpa, 3 dpa, 1 wpa and 2 wpa. Error bars represent the standard deviation (p < 0.05). Scale bars, 400 μm in (**a**), (**d**), (**f**) and (**i**); 100 μm in (**b**), (**c**), (**e**), (**g**) and (**h**).

The occurrence of the gecko blastema begins around 3–4 days between retracted spinal cord and overlying clot after tail amputation^4^. There are two models for blastemal cells to acquire positional identity. One is that the cells experience different concentrations of RA along the axis, and this sets the appropriate initial level. The other is that the molecule with positional identity is expressed in a stable gradient along the axis in the cells that are precursors of blastemal cells, and that after amputation the blastemal cells inherit this level of expression^12^. It seems unlikely for the tail blastemal cells to acquire positional identity in the second model, as the transcriptional level of *CD59* remains unchanged in the intact tail^18^. To examine the initial expression of CD59 in the regenerating stump, horseradish peroxidase (HRP) staining, instead of immunofluorescence, was performed to detect the tissue distribution to avoid autofluorescence in epidermis and other tissues. We raised rabbit polyclonal antibody against the peptide RESYNCWKYSQCDGK that corresponds to residues 55–69 of the mature protein. At 1 day postamputation (dpa), HRP staining was detectable at the healed injury site capped with blood clot and migrating cells from epidermis. The positive signals were also observed at the terminus of the amputated spinal cord (Fig. 1A–C). At 3 and 7 dpa, the blastemal cells between retracted spinal cord and overlying wound epithelium were CD59-positive, and HRP staining was still present at the terminus of the amputated spinal cord and wound epithelium (Fig. 1D–H). It is worthy to note that the positive signals are not restricted to the cell surfaces, indicative of the presence of soluble CD59 (Fig. 1A–H). Western blots of tissues from both spinal cords and regenerates, as well as the negative controls, excluded the non-specific reaction of the antibody (Fig. 1I,J). Real time PCR revealed that the expression of gecko *CD59* in the blastema was strongly upregulated at 3 dpa, concomitant with the appearance of the early blastema, but decreased at 1 week. A significant increase of *CD59* expression was also detected at 2 weeks (Fig. 1K). These data suggest that the occurrence of CD59 is earlier than that of the blastema in diverse cell types.

### Full-length of CD59 is required for positional identity

**Figure 2:**
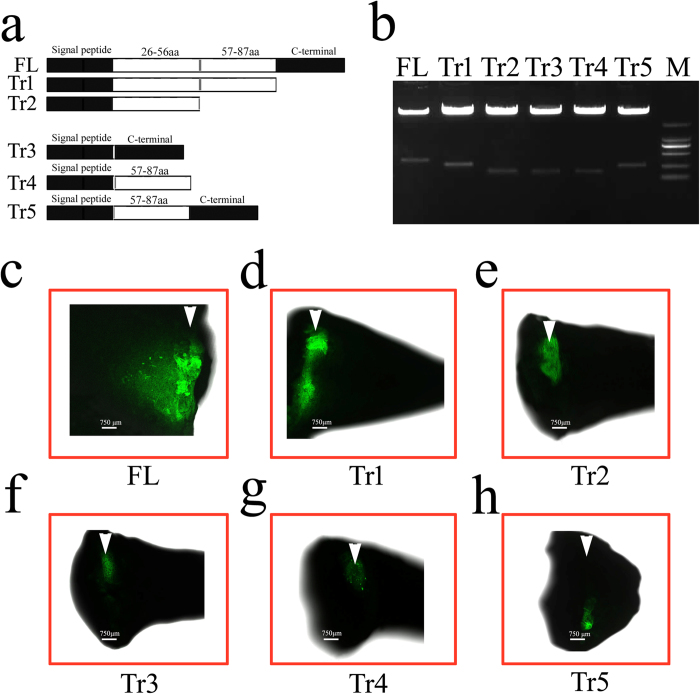
Overexpression of full-length CD59 and different truncates in the blastemal cells. (**a**) Schematic diagram of CD59 truncates; (**b**) Enzyme digestion of recombinant plasmid constructs for CD59 truncates using Xho I and BamH I enzymes; (**c**–**h**) The stumps at 2 wpa were electroporated with plasmids of full-length CD59 or different truncates. Arrowhead indicates the plasmid-injected site. Scale bars, 750 μm.

CD59 has been found to mediate the positional identity in the blastema by proximalization of blastemal cells when overexpressed^18^. To determine the potential active sequence(s) involved in this function, we created different truncates by sequentially deleting 30 residues after the signal peptide (Fig. 2A–B), in consideration of the dispersed active sites in the full sequence^21^. These truncates were cloned into the pEGFP-N3 vector, and confirmed by enzyme digestion and DNA sequencing (Fig. 2B). When plasmids containing full-length CD59 protein were electroporated into the blastemal cells for 3 days, the transfected cells shifted proximally (Fig. 2C), in agreement with our previous findings^18^. While electroporation with various CD59 truncates, the marked cells remained in the region of plasmids-microinjected sites (Fig. 2D–H). These results indicate that full-length CD59 is required for positional identity.

### Protection of CHO cells from complement-mediated lysis by CD59

**Figure 3:**
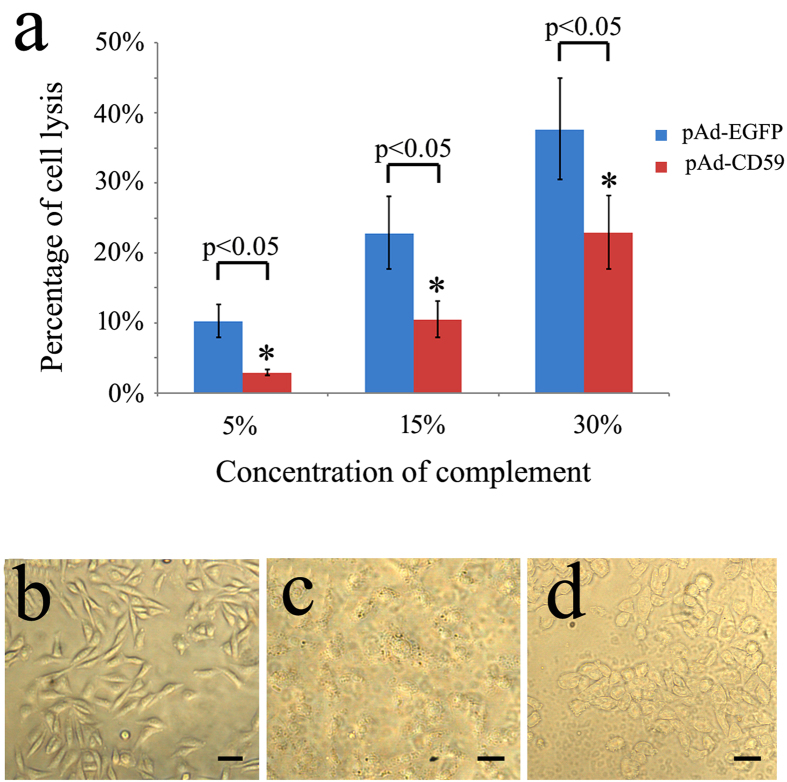
Determination of complement-mediated CHO cell lysis following pAd-CD59 transfection. (**a**) Percentage of cell lysis was determined using MTT assay following transfection with pAd-EGFP or pAd-CD59 for 36 h, and stimulation with rabbit complement at concentration of 5%, 15% and 30%, respectively; (**b**) Showing CHO cells cultured in DMEM/F12 supplemented with 10% FBS; (**c**) and (**d**) Showing CHO cells stimulated with complement at concentration of 30% following tranfection of with pAd-EGFP (**c**) or pAd-CD59 (**d**). Error bars represent the standard deviation (p < 0.05). Scale bars, 50 μm.

It is worthy to mention that the expression of CD59 in the amputated tail occurs before the appearance of blastema (3–4 dpa), and is detectable at the terminus of the injured spinal cord (Fig. 1). This observation implies additional physiological roles for CD59 in the neurons. Upregulation of neuronal CD59 was found to protect neurons from complement-mediated degeneration^22^. We wished to uncover its function in immune regulation, which might be required in the tail regeneration. Antibody-sensitized CHO cells are efficiently lysed by rabbit serum complement (Fig. 3A–C). However, the transfection with pAd-CD59 appeared to protect the CHO cells from complement-mediated lysis (Fig. 3A,D). These data indicate that gecko CD59 functions on both positional identity and immune regulation during the tail regeneration.

### Intervention of CD59 results in the abnormal patterning of cartilage

**Figure 4:**
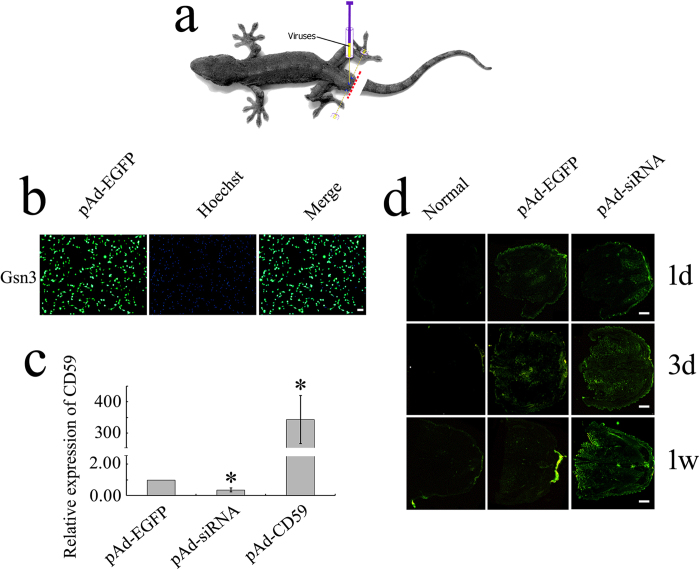
Transfection of pAd-CD59 or pAd-siRNA adenovirus in the regenerating tail stump of gecko. (**a**) Schematic diagram of adenovirus injection in the tail stump. Xue Bai took the photograph of the gecko; (**b**) The determination of pAd-EGFP adenovirus transfection efficiency in the gecko Gsn3 cell line; (**c**) Analysis of *CD59* expression by real-time PCR following transfection with pAd-CD59 or pAd-siRNA adenovirus for 24 h; (**d**) Section observation of adenovirus transfection in the regenerating tail stump of 1 dpa, 3 dpa and 1 wpa by green fluorescence of pAd-EGFP or pAd-siRNA constructs. Scale bars, 100 μm in (**b**), 400 μm in (**d**).

**Figure 5:**
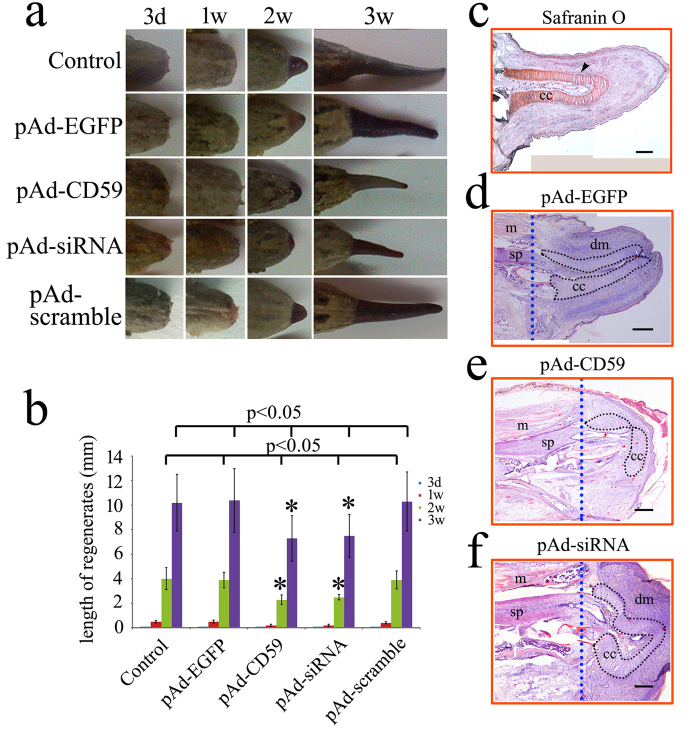
Effects of CD59 intervention on the regenerating tail. (**a**) Phenotype of CD59 overexpression or siRNA knockdown following injection of pAd-CD59 or pAd-siRNA adenovirus for 3 dpa, 1 wpa, 2 wpa and 3 wpa; (**b**) Statistic analysis of regenerating tails following adenovirus transfection; (**c**) Safranin O staining of regenerate at 2 wpa; (**d**–**f**) HE staining of longitudinal sections for the stumps injected with pAd-EGFP, pAd-CD59 or pAd-siRNA adenovirus for 2 wpa; m, muscle; sp, spinal cord; dm, differentiating muscle; cc, cartilaginous cone. Scale bars, 750 μm in (**c**) and (**d**); 400 μm in (**e**) and (**f**).

To ascertain the regulatory effects of CD59 on the patterning of tail regeneration, a total of 9 μl pAd-EGFP, pAd-CD59, pAd-siRNA or pAd-scramble siRNA adenovirus in three aliquots was immediately injected into the area around the vertebrae of the amputated tail, 2–3 mm anteriorly to the plane (Fig. 4A). The transfection efficiency was assayed by the intensity of GFP fluorescence in the gecko oligodendrocyte cell line Gsn3 (Fig. 4B,C) and in the sections of the regenerates (Fig. 4D), except for those of pAd-CD59. Real-time PCR analysis demonstrated that the expression level of *CD59* was either upregulated (pAd-CD59) or downregulated (pAd-siRNA) following adenovirus transfection in the Gsn3 for 24 h (Fig. 4C), confirming the effectiveness of *in vivo* genetic intervention. The regenerating tail transfected with pAd-CD59 or pAd-siRNA displayed a phenotype of shortened length following 2 weeks postamputation (wpa), in comparison to those with the injection of vehicle, pAd-EGFP or pAd-scramble siRNA (Fig. 5A,B). The wound healing in the pAd-CD59 or pAd-siRNA transfected animals was delayed until 2 dpa (Data not shown). At 3 wpa, the tail length in CD59-intervened group is approximately 70% of those in the non-intervened (Fig. 5A,B). To elucidate the defect element(s) in the regenerates, HE staining was performed in the sections of 2 wpa tails. Safranin O staining showed a well-defined cartilaginous cone (Fig. 5C). Obviously, the cartilaginous cone, which is involved in the formation of cartilage, is malformed following CD59 intervention (Fig. 5D–F). These data indicate that intervention of CD59 results in the abnormal patterning of cartilage.

### Influence of CD59 on the differentiation of blastemal cells to chondrocytes

**Figure 6:**
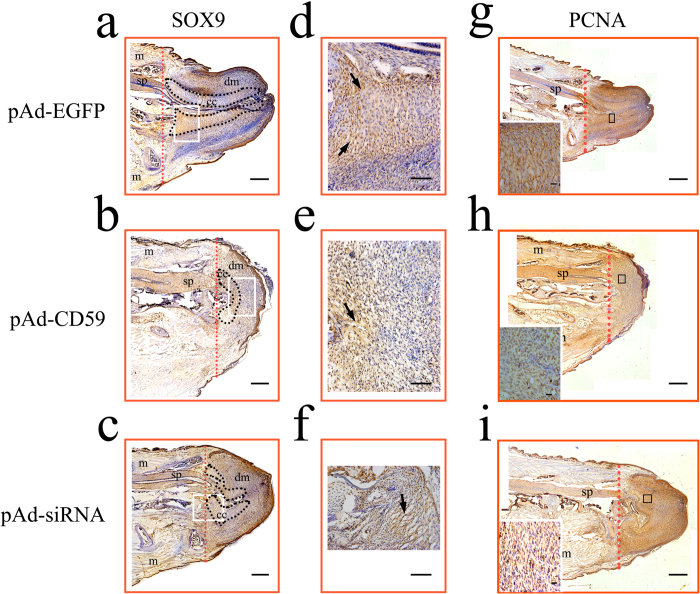
Localization of SOX9- and PCNA-positive cells in the regenerating tails following CD59 intervention. (**a**–**c**) SOX9 staining of longitudinal sections for the stumps injected with pAd-EGFP, pAd-CD59 or pAd-siRNA adenovirus for 2 wpa, respectively; (**d**), (**e**) and (**f**) indicate higher magnification of the boxed regions in (**a**), (**b**) and (**c**), respectively; (**g**–**i**) PCNA staining of longitudinal sections for the stumps injected with pAd-EGFP, pAd-CD59 or pAd-siRNA adenovirus for 2 wpa, respectively. Inserts are the higher magnification of the boxed regions in (**g**–**i**). m, muscle; sp, spinal cord; dm, differentiating muscle; cc, cartilaginous cone. Scale bars, Scale bars, 750 μm in (**a**) and (**g**); 400 μm in (**b**), (**c**), (**h**) and (**i**); 100 μm in (**d**–**f**); 20 μm in inserts.

To further clarify the influence of CD59 on the chondrogenesis, we observed the localization of SOX9, the transcription factor that functions as a master regulatory factor in the chondrocyte differentiation^23^. The blastemal cells displayed negative staining of SOX9 at stump of 1 wpa (data not shown). Instead, cartilage precursor cells with SOX9-positive staining were observed in the cartilaginous cone at 2 wpa (Fig. 6A,D). Overexpression or siRNA knockdown of CD59 with adenovirus both decreased the number of SOX9-positive cells (Fig. 6B,C,E,F). To exclude the possibility that CD59 affects the proliferation of all blastemal cells, the proliferating cell nuclear antigen (PCNA) staining was performed following CD59 intervention. Transfection of pAd-CD59 or pAd-siRNA demonstrated that the number of proliferating cells showed no significant difference at the stump of 2 wpa (Fig. 6G–I). These results indicate that CD59 mediates the differentiation of blastemal cells to chondrocytes.

### Colocalization of CD59 with SOX9-positive cells

**Figure 7:**
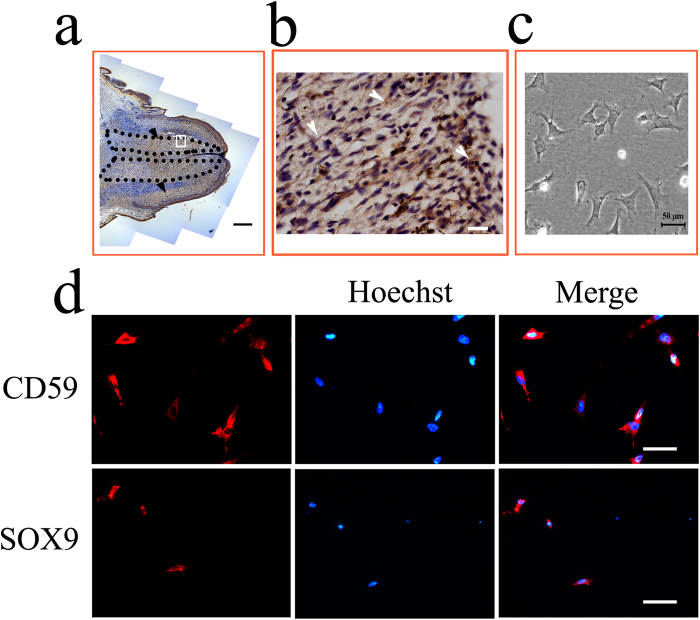
Localization of CD59 and SOX9 in the blastemal cells. (**a**) Localization of CD59 in the blastemal cells of regenerating tail at 2 wpa; (**b**) Higher magnification of the boxed regions in (**a**). Arrowheads indicate CD59-positive signals in the cell surface; (**c**) The light micrograph of blastemal cells cultured from regenerating tail at 2 wpa; (**d**) Localization of CD59 and SOX9 in the blastemal cells cultured from regenerating tail at 2 wpa. Scale bars, 750 μm in (**a**); 50 μm in (**c**) and (**d**); 20 μm in (**b**).

To address whether CD59 colocalizes with SOX9-positive cells at the stages of blastemal cell differentiation, we compared the tissue distribution of CD59 and SOX9 at 2 wpa. CD59-positive staining was detected in the distinct cartilaginous cone (Fig. 7A,B), with an overlap with SOX9-positive cells (Fig. 6A). We further cultured the blastemal cells from stump of 2 wpa (Fig. 7C), and validated with the stem cell marker SOX2 (data not shown). All the blastemal cells displayed CD59-positive staining, and nearly 60% of these cells were SOX9-positive (Fig. 7D). These results reveal that CD59 likely regulates the differentiation of blastemal cell to chondrocytes.

### Cartilage formation-related genes are regulated by CD59

**Figure 8:**
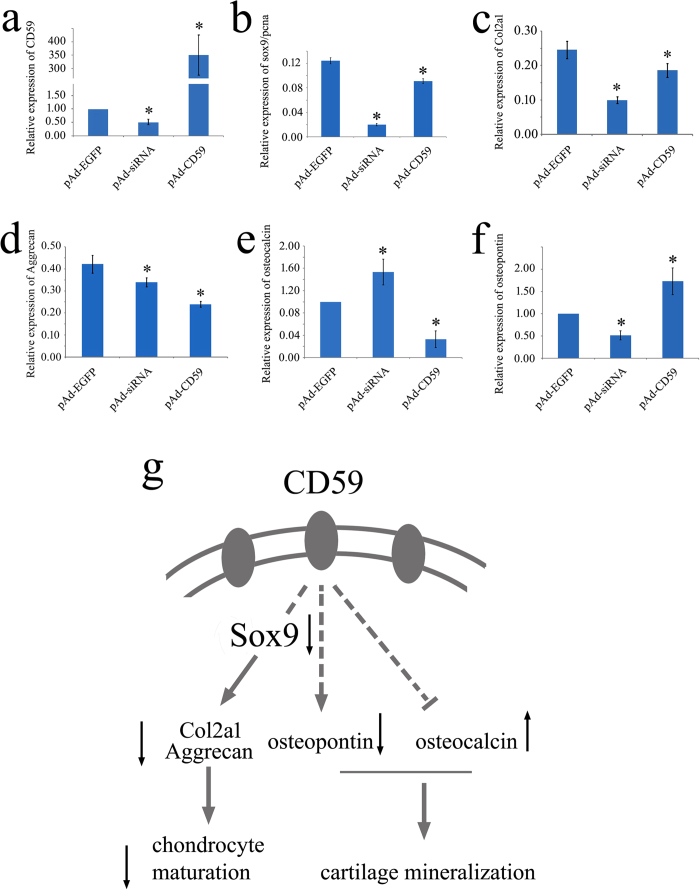
Expression of cartilage formation-related genes following CD59 intervention in the blastemal cells of 2 wpa. (**a**–**f**) Analysis of *CD59* (**a**), relative expression of *sox9*/*pcna* (**b**), *Col2a1* (**c**), *Aggrecan* (**d**), *osteocalcin* (**e**), and *osteopontin* expression (**f**) by real-time PCR following transfection with pAd-CD59 or pAd-siRNA adenovirus for 24 h; (**g**) Illustration of CD59 regulation on the differentiation of blastemal cells to chondrocytes, and cartilage formation. Error bars represent the standard deviation (p < 0.05).

The cartilage regeneration of gecko tail has shown bone-remodeling activities, with endochondral ossification in the proximal cartilage tube and subsequent mineralization (calcification) in the distal tube^17^. To unveil the underlying molecular mechanisms of CD59 in regulating chondrogenesis following CD59 intervention in the blastemal cells, we examined the expression levels of two genes related to cartilage formation: collagen, type II, α1 (*Col2a1*) and *Aggrecan*, the cartilage specific markers downstream of Sox9. We also detected the expression of *osteocalcin* and *osteopontin*. Osteocalcin is a small g-carboxyglutamate protein preferentially expressed by osteoblasts and binds to calcium ions. The role of osteocalcin in bone is to regulate bone mineralization and bone turnover^24^. The protein Osteopontin (OPN) is a secreted phosphoglycoprotein expressed in various cell types including osteoclasts, osteoblasts and chondrocytes, and is involved in the bone-remodeling process^25^. Our results showed that the expression levels of both *Col2a1* and *Aggrecan* were significantly downregulated following the transfection of pAd-CD59 or pAd-siRNA in blastemal cells for 24 h, similar to that of *Sox9* (Fig. 8B–D). CD59 was observed negatively regulating the expression of *osteocalcin* (Fig. 8E), and positively regulating the expression of *osteopontin* (Fig. 8F). Our results indicate that CD59 not only affects the differentiation of blastemal cells to chondrocytes, but also subsequent bone-remodeling through regulation of cartilage/bone formation-related genes.

## Discussion

It is very difficult to genetically manipulate gecko, owing to oviposition at stages 28–29 (*in vivo* development) and hatching at stage 42^26^. This limits, to some extent, functional investigation of several important regenerative-related molecules *in vivo*. In this study, we have successfully established an approach for *in vivo* genetic manipulation in the non-mammalian amniote. Injection of pAd-CD59 or pAd-siRNA adenovirus displayed valid transfection in the blastemal cells, while lentiviruses were found to be less efficient in the gecko (data not shown). Although adenoviral DNA cannot be integrated into the genome of the host cells, the vectors are still detectable at 7 dpa (Fig. 4D), lasting until the appearance of blastemal cells.

Gecko CD59, similar to those in mammals, protects cells from complement-mediated lysis. Following gecko tail amputation, CD59 was immediately upregulated in the injured spinal cord retracting within neural canal (Fig. 1C). It is well established that the central nervous system contains and synthesizes many components of the immune system. Neurons, astrocytes, microglia and oligodendroglia were shown to produce all the complement components of both alternative and classical pathways, and also the proteins involved in the terminal pathway forming membrane attack complex (MAC)^22^,^27^,^28^,^29^,^30^. Neurons of mammals express only low levels of CD59^31^, thus vulnerable to MAC lysis, resulting in neuronal death or degeneration post injury^22^. In contrast, several regeneration-competent vertebrates are able to spontaneously regenerate the spinal cord following injury, and re-innervate target elements without neuronal degeneration occurring in mammalian counterparts^3^,^15^, suggesting that upregulation of CD59 might play a role to protect the neuron from lysis.

The origin and differentiation of blastemal cells have been the subjects of intensive studies for several decades. By specifically labeling most major limb cell types in the axolotl, or by directly grafting a specified GFP^+^ limb tissue to an unlabeled host, Kragl *et al.* have shown that regenerating cell types mostly retain their developmental identity as they transition through the blastemal stage, and normally do not create diverse cell types^8^. An important question is how these blastemal cells acquire positional identity, thereafter are regulated. The dermal layer of skin is a major contributor to the blastema, and has been proposed to have potent patterning effects on regeneration^32^,^33^,^34^. Kragl *et al.* have confirmed that dermis covering the amputated limb is capable of forming cartilage and tendons but not muscle, owing to the common origin of limb dermis and cartilage in the lateral plate mesoderm^8^. These observations suggest that dermal tissue contributes to the formation of cartilage progenitor cells. Retinoic acid (RA) signaling is immediately initiated in the wound epithelium (to form different layers of skin) following appendages amputation^35^, through which the precise patterning of blastema was established. The appearance of CD59 at the healed injury site, and subsequently at the wound epithelium, suggests that the cartilage progenitor cells derived from wound epithelium acquire positional identity under the regulation of RA signaling.

Intervention of CD59 expression resulted in the downregulation of *Sox9*, the transcription factor that is necessary for stem cells to execute the chondrogenic differentiation program^17^. Two chondrocyte-specific extracellular matrix proteins, Col2a1 and Aggrecan, were downregulated under the control of Sox9, indicative of the regulatory function of CD59 on chondrocyte differentiation. The cartilage regeneration of lizard tail includes two distinct mineralization events. The non-collagenous proteins, such as osteocalcin and osteopontin, have been reported in the regulation of bone turnover and mineralization. Currently osteocalcin has been shown to upregulate the expression of osteochondrogenic transcription factors SOX9 via hypoxia-inducible factor 1α signaling in vascular smooth muscle cells, as well as a shift in cellular metabolism toward glycolysis, suggesting a critical function in chondrogenesis^36^. Osteopontin, a secreted protein, is developmentally expressed during gastrulation in the notochord and the embryonic/maternal interface, and later in regions of cartilage condensation and bone formation^37^,^38^. One major physiological function of osteopontin is to control biomineralization, and osteopontin^−/−^ bones are hypermineralized and more fragile than those from wild-type mice^39^. The observation that CD59 has positive regulatory effect on osteopontin and negative regulatory effect on osteocalcin suggests that a balanced expression level of CD59 is indispensible for the spatial-temporal mineralization of the new cartilage during the tail regeneration. These would partly explain why changes of positional identity in blastemal cells lead to abnormal regeneration of appendages^40^.

CD59 molecules are widely retained from fish onwards in the phylogeny, and its functions on immune regulation have been extensively reported from the basal vertebrates to mammals^19^,^41^. One of the CD59/Ly6 protein family members, the Prod1, has been found to regulate proximodistal cell identity in amphibian^11^,^13^,^14^,^40^. Subsequently, it was found that the Prod1 ligand, a newt anterior gradient protein (nAG), was able to rescue limb regeneration in denervated newt limbs, suggesting that Prod1 is sufficient to regulate the regenerative response^12^. We previously revealed that CD59 in gecko was involved in the regulation of positional identity during the tail regeneration^18^, suggesting that proteins of CD59/Ly6 family have been adapted by various vertebrate lineages to regulate patterning during the appendage regeneration^7^. The finding that CD59 regulates cartilage patterning by decreasing the differentiation of blastemal cells to chondrocytes has provided a novel molecular mechanism for chondrogenesis. By mediating both immune and regenerative functions in the regenerative model, CD59 functions as a key regulator linking immune response and regenerative capability.

In summary, CD59 affects the differentiation of blastemal cells to chondrocytes *via* Sox9 signal pathway, and subsequent bone-remodeling via the regulation of cartilage/bone formation-related genes *osteocalcin* and *osteopontin* (Fig. 8G). In addition, CD59 mediates immune regulation by protecting cells from complement-mediated lysis. Thus, CD59 is an important linker between immune response and regenerative capability.

## Methods

### Animals

Adult *Gekko japonicus* were used as described by Wang *et al.*^3^. Briefly, adult animals were fed *ad libitum* with mealworms and housed in an air-conditioned room with a controlled temperature (22–25 °C) and saturated humidity. Anesthesia was induced by cooling the animals on ice prior to tail amputation. Amputation was performed at the sixth caudal vertebra, identified based on the special tissue structure present at that position^4^, by placing a slipknot of nylon thread and pulling gently until the tail was detached, thus mimicking the autotomy undergoing for natural defense.

All experiments were conducted in accordance with guidelines established by the NIH, found in *Guide for the Care and Use of Laboratory Animal* (1985), and by the Society for Neuroscience, found in *Guidelines for the Use of Animals in Neuroscience Research*. The experiments were approved according to the Animal Care and Use Committee of Nantong University and the Jiangsu Province Animal Care Ethics Committee. All geckos (n = 15) were anaesthetized on ice prior to euthanatizing.

### Blastemal cell culture

Gecko tail regenerates (generally 5–10) were harvested at 1 week or 2 weeks post amputation, and the wound epidermis was removed. The tissue was cut into pieces of nearly 1 mm^3^, and digested with 0.05% trypsin (Boehringer, France) for 12–24 h at 8 °C, and dissociated. Following wash with PBS (pH 7.2), they were transferred to collagen-coated microwells and added with Dulbecco’s Modified Eagles Medium (DMEM, Gibco) and F12 (1:1) containing 1% FBS and 1% penicillin/streptomycin (Invitrogen). The cells were cultured for 3–5 days at 30 °C before ongoing experiments.

### Production of CD59 overexpression and siRNA adenovirus

CD59 overexpression (pAd-CD59) and siRNA (pAd-siRNA) adenovirus were produced in Invitrogen Biotech Co. (Shanghai), according to the manufacturer’s procedures. Briefly, the recombinant sequence of CD59 was amplified by recombinant primer pairs ATTB1-CD59: 5′- GGGGACAAGTTTGTACAAAAAAGCAGGCTTCGCCACCATGAAGTGTCTCTTGATCACTGTTGC -3′ and ATTB2-CD59: 5′- GGGGACCACTTTGTACAAGAAAGCTGGGTC TCAGATTAAAAGCAGTGTGGTCAGAAAAG -3′ in the volume of 50 μl reaction system (1 μl pcDNA3.1-CD59 template, 5 μl 10 × AccuPrime pfx Reaction mix, 5 μl 10 × enhancer, 0.5 μl AccuPrime pfx polymerase, 1 μl each primer, and 36.5 H_2_O). The fragment was further purified and cloned into pDONR221 vector in the BP recombination reaction system (2 μl CD59 cDNA, 1 μl pDONR221 vector, 2 μl BP clonase II enzyme mix, 5 μl TE Buffer, pH8.0). The BP recombinant vector was subsequently constructed into adenovirus vector pAd CMV/V5-DEST using LR recombination reaction (1 μl BP recombinant vector, 1 μl pAd CMV/V5-DEST, 2 μl LR clonase II enzyme mix, 6 μl TE Buffer, pH8.0). Finally, the adenovirus recombinant vector was linearized by Pac I enzyme, packaged into the 293A cells, and the adenovirus was collected from lysed cells. For the production of pAd-siRNA adenovirus, the same procedure was referred except the synthesized siRNA of CD59 was cloned into pcDNA6.2-GW/miR vector (Invitrogen, Shanghai). The viral titer is 3.8 × 10^10^ ifu/ml for pAd-CD59, and 3.25 × 10^10^ ifu/ml for pAd-siRNA.

### Hematoxylin-eosin (HE) staining and immunohistochemistry

The regenerating tails were harvested, post-fixed and sectioned. Sections were counterstained with HE, dehydrated in a graded ethanol series, cleared in xylene, and coverslipped with HSR solution (Sysmex; Kobe, Japan).

For the immunohistochemistry, sections were allowed to incubate with ployclonal rabbit anti-gecko CD59 (1:100 dilution, BIOSS), polyclonal rabbit anti-bovine Sox9 antibody (1:200 dilution, Abcam), or rabbit polyclonal to PCNA antibody (1:200 dilution, Abcam) at 4 °C for 24 h. The sections were treated with SPlink Detection Kits (ZSGB-BIO), then colouration with 3,3-diaminobenzidin (DAB) at room temperature without light for 5 min. Sections were washed with the distilled water, dehydrated in a graded ethanol series, and cleared in xylene before mounting with neutral gums. The staining was observed under microscope (Leica, Heidelberg, Germany).

### Quantitative real-time polymerase chain reaction (Q-PCR)

Total RNA was prepared with Trizol (Gibco, USA) from regenerates of 20 geckos amputated from the sixth caudal vertebra at 1 day, 3 days, 1 week and 2 weeks, respectively. Total RNAs were also extracted from cultured blastemal cells treated with pAd-CD59 or pAd-siRNA at desired stages. For Q-PCR examination of related genes transcriptional expression, the first-strand cDNA was synthesized using Omniscript Reverse Transcription Kit (QIAGEN) in a 20 μL reaction system containing 2 μg total RNA, 0.2 U/μL M-MLV reverse transcriptase, 0.5 mM dNTP mix, 1 μM Oligo-dT primer. The cDNA was diluted 1:5 before use in Q-PCR assays. The sequence-specific primers and Taqman probe were designed and synthesized by Invitrogen (Shanghai, China). Primer pair and probe for *CD59*: forward primer 5′-CTG GGA GAA CAA AGA GAG TCC T-3′, reverse primer 5′-AAAGATGCTATG CTGAGGGAGA-3′; for *Col2a1*: forward primer 5′-GAAGAGGGGTGACTACTGGATT-3′, reverse primer 5′-CAAACCAGATGTGCTTCTTCTC-3′; for *Aggrecan*: forward primer 5′- CTACATTGACACACTGGAGCA -3′, reverse primer 5′- ATCAGTAGGAATGGCAGGGTA -3′; for *osteocalcin*: forward primer 5′-TGGAACACGCTCCCAAACGA-3′, reverse primer 5′-AGACGCCCACTGCCTCAACC-3′; for *osteopontin*: forward primer 5′-AGCAACAAACCCTTCT-3′, reverse primer 5′-AGTCATCCGCCTCACC-3′. Q-PCR reactions were performed in a final volume of 20 μl (1 μl cDNA template and 19 μl Q-PCR reaction buffer containing 2.5 mmol/L MgCl2, 0.2 mmol/L dNTPs, anti-sense and sense primers 0.5 μmol/L, taqman probe 0.4 μmol/L, DNA polymerase 0.2 μl and 1 × DNA polymerase buffer). The Rotor-Gene 5 software (Corbett Research, Rotor-Gene, Australia) was used for real-time PCR analysis. Reactions were processed using one initial denaturation cycle at 93 °C for 2 min followed by 40 cycles of 93 °C for 30 sec, 56 °C for 30 sec and 72 °C for 30 sec. Fluorescence was recorded during each annealing step. At the end of each PCR run, data were automatically analyzed by the system and amplification plots obtained. CD59 full-length plasmid was used to prepare standard curves and used as a specificity control for real-time PCR. The expression levels were normalized to an endogenous EF-1α cDNA using forward primer 5′-CCTTCAAATATGCCTGGGT-3′, reverse primer 5′-CAGCACAGTCAG CTTGAGAG-3′ and taqman probe 5′-TTGGACAAGCTGAAGGCAGAACGTG-3′. In addition, a negative control without the first-strand cDNA was also performed.

### Electroporation of CD59 truncates in blastema

A total of five CD59 truncates were constructed by PCR techniques using corresponding primer pairs: 5′- ccCTCGAGCTatgaagtgtctcttgatcactgt -3′ and 5′- cgGGATCCtgacttgcagcac ctaaac -3′; 5′- ccCTCGAGCTatgaagtgtctcttgatcactgt -3′ and 5′- ccGGATCCctctctttgttcaccc agg -3′; 5′- ccCTCGAGCTatgaagtgtctcttgatcactgt -3′ and 5′- cgGAATTCgtcataacactttatgaca -3′; 5′- cgGAATTCgacttgtgtaatagtagtccat -3′ and 5′- cgGGATCCgattaaaagcagtgtggtcag -3′; 5′- cgGAATTCtcctataactgctggaaata -3′ and 5′- cgGGATCCtgacttgcagcacctaaac -3′; 5′- cgGAAT TCtcctataactgctggaaata -3′ and 5′- cgGGATCCgattaaaagcagtgtggtcag -3′. Then the fragments were subjected to restriction endonuclease BamH I, EcoR or Xho I, subsequently to ligase for different truncates. Tail blastemas were electroporated at 14 days post-amputation. Animals were anesthetized on ice and immobilized. Three μl of each CD59 truncate constructed in pEGFP-N3 vector was injected into the middle of the blastema using a microinjector. External pulses were then applied using electrotransfer (ECM 830, Neucleofecto), 200 V, and 50 ms, two times with interval of 1 second. Three days post-electroporation, the blastemas were cut and imaged under two-photon laser scanning fluorescence microscopy (Leica sp5).

### Complement lysis assays

Chinese hamster ovary (CHO, Chinese Academy of Sciences, Shanghai Institutes for Biological Sciences Cell Resource Center) cells were grown in DMEM/F12 supplemented with 10% FBS in a 37 °C humidified incubator with 5% CO_2_. Cells were resuspended to 2 × 10^5^/ml, and seeded to a 24-well plate in 500 μl medium for overnight. Then, adenovirus solution of pAd-EGFP or pAd-CD59 was added to the culture at multiplicity of infection (MOI) of 10. After 36 h post transfection, cells were resuspended and adjusted to the density of 2 × 10^4^/ml. They were seeded and cultured in a 96-well plate in 100 μl medium until to the confluency of 80%.

Cells were preincubated in serum-free medium for 2 h, washed twice with HBSS before addition of rabbit anti-CHO cell membrane antiserum at 37 °C for half hour. After another twice washes in HBSS, cells were incubated in 50 μl of serial dilutions of the rabbit complement at 37 °C for half hour. Then, the supernatant was removed from the culture, and 100 μl mixture of 3-(4,5-dimethylthiazol-2-yl)-2,5-diphenyltetrazolium bromide (MTT) (5 mg/ml) and DMEM/F12 (1:4) was added to each well. Following incubation for 4 h, the supernatant was replaced with 100μl DMSO, and oscillated for 10 min. Percentage of cell lysis was determined using MTT assay by detection of optical density (OD) values at 490 nm.

### Statistical analysis

Statistical significance of differences between groups was analyzed by unpaired t test or by one-way analysis of variance (ANOVA) with SPSS 15.0 (SPSS, Chicago, IL, USA) when more than two groups were compared. Statistical significance was set at P < 0.05.

## Additional Information

**How to cite this article**: Bai, X. *et al.* CD59 mediates cartilage patterning during spontaneous tail regeneration. *Sci. Rep.* **5**, 12798; doi: 10.1038/srep12798 (2015).
